# Accelerated hypofractionated radiotherapy plus chemotherapy for inoperable locally advanced non-small-cell lung cancer: final results of a prospective phase-II trial with a long-term follow-up

**DOI:** 10.1186/s13014-019-1317-x

**Published:** 2019-06-24

**Authors:** Elisabetta Parisi, Giovenzio Genestreti, Anna Sarnelli, Giulia Ghigi, Donatella Arpa, Marco Angelo Burgio, Giampaolo Gavelli, Alice Rossi, Emanuela Scarpi, Manuela Monti, Anna Tesei, Rolando Polico, Antonino Romeo

**Affiliations:** 1 Radiotherapy Unit, Istituto Scientifico Romagnolo per lo Studio e la Cura dei Tumori (IRST) IRCCS, Meldola, Italy; 2 Department of Medical Oncology, Institute of Neurological Sciences, Bellaria Hospital IRCCS, Meldola, Italy; 3 Medical Physics Unit, Istituto Scientifico Romagnolo per lo Studio e la Cura dei Tumori (IRST) IRCCS, Meldola, Italy; 4 Department of Medical Oncology, Istituto Scientifico Romagnolo per lo Studio e la Cura dei Tumori (IRST) IRCCS, Meldola, Italy; 5 Radiology Unit, Istituto Scientifico Romagnolo per lo Studio e la Cura dei Tumori (IRST) IRCCS, Meldola, Italy; 6 Unit of Biostatistics and Clinical Trials, Istituto Scientifico Romagnolo per lo Studio e la Cura dei Tumori (IRST) IRCCS, Meldola, Italy; 7 Biosciences Laboratory, Istituto Scientifico Romagnolo per lo Studio e la Cura dei Tumori (IRST) IRCCS, Meldola, Italy

**Keywords:** Inoperable locally advanced non-small-cell lung cancer, Accelerated hypofractionation, Intensity modulated arc therapy (IMAT), Radiotherapy and chemotherapy

## Abstract

**Background:**

Concurrent chemotherapy and radiation using conventional fractionation is the standard treatment for inoperable, locally advanced non-small-cell lung cancer (NSCLC). We tested accelerated hypofractionated radiotherapy (AHR) and chemotherapy for the treatment of locally advanced NSCLC.

**Methods:**

Eligible patients with locally advanced NSCLC were treated with induction chemotherapy (cisplatin and docetaxel), followed by AHR using tomotherapy and consolidation chemotherapy. The prescribed doses were 30 Gy/5 daily fractions at the reference isodose (60–70%) to the tumor, and 25 Gy/5 daily fractions to the clinically involved lymph nodes. The primary end-point was response rate (RR); the secondary end-points were acute and late side-effects, local progression-free survival (PFS), metastasis-free survival (MFS) and overall survival (OS). This trial closed before the first planned interim analysis due to poor accrual.

**Results:**

From January 2009 to January 2012, 17 of the 23 enrolled patients were evaluable. Treatment yielded an overall RR of 82%. Median follow-up was 87 months (range: 6–87), local PFS was 19.8 months (95% CI 9.7 - not reached), MFS was 9.7 months (95% CI 5.8–46.0) and OS was 23 months (95% CI 8.4–48.4). 70% of patients experienced acute G4 neutropenia, 24% G4 leukopenia, 24% G3 paresthesia, 4% G3 cardiac arrythmia, 4% underwent death after chemotherapy. Late toxicity was represented by 24% dyspnea G3.

**Conclusions:**

AHR combined with chemotherapy is feasible with no severe side-effects, and it appears highly acceptable by patients.

**Trial registration:**

This study is registered with the EudractCT registration 2008-006525-14. Registered on 9 December 2008.

## Introduction

Non-small-cell lung cancer (NSCLC) represents the most common cause of death from cancer worldwide, in particular in Europe and the USA [1]. Tobacco use is the main risk factor for lung cancer, with a high rate of other pulmonary tumors related to the effects of smoking [2].

The majority of patients present with locally advanced or metastatic disease at diagnosis [3]. In this setting, chemotherapy and concomitant radiotherapy represent the gold standard treatment. Some studies [4, 5] have compared sequential with concomitant chemoradiotherapy with conventional fractionation showing the superiority of the latter, although with a higher rate of acute and chronic side-effects. However, poor long-term results are distinctive of concomitant chemoradiotherapy in the treatment of locally advanced NSCLC.

The integration of chemotherapy and radiotherapy has posed many problems about potential side-effects. Radiotherapy has shown better effectiveness in locoregional control on increased dose [6], as pointed out by the Radiation Therapy Oncology Group (RTOG) 73–01 landmark trial [7]. However, some dose escalation trials have reported that a higher locoregional control is not correlated to survival benefits, except for the cut-off of 60 Gy with 3-dimensional conformal radiotherapy (3D-CRT) [8–10]. RTOG 0617, compared 60 Gy vs. 74 Gy both with chemotherapy (carboplatin and paclitaxel) and reported a median survival of 29 months in patients receiving 60 Gy versus 20 months in patients receiving 74 Gy. Furthermore, the 74 Gy arm showed an increased risk of death leading to an early closure of the trial [11].

Attempts to increase the total dose of conventional treatments and the use of hyperfractionated treatment have yielded no substantial difference from previous experiences. Arimoto et al. [12] observed interesting efficacy results in early-stage peripheral lung tumor using accelerated hypofractionated radiotherapy (AHR). Polico et al. [13] reported that AHR is efficacious, feasible and safe also for locally advanced disease, i.e. lung tumor and mediastinal nodes. The only possibility to improve treatment outcomes for locally advanced non small cell lung cancer is to increase local control and avoid metastatic spread.

The aim of our study was to increase local control through AHR and sequential chemotherapy for patients with locally advanced NSCLC. We analyzed RR and down-staging in NSCLC and involved mediastinal lymph nodes as primary endpoint. Secondary endpoints were acute and late side-effects, local progression-free survival (PFS), metastasis-free survival (MFS) and overall survival (OS).

## Methods and materials

We conducted a prospective phase-II clinical trial enrolling cyto-histologically proven, locally advanced NSCLC patients. All patient characteristics are shown in Table 1.

**Table 1:** Patient characteristics: (y) years; (n) number

Characteristic	Value *y, n* (%)
*Age (y)*	
Median	58
Range	43–69
*Gender* (*n*)	
Male	13 (76)
Female	4 (24)
*Histologic subtype* (*n*)	
Adenocarcinoma	10 (59)
Squamous cell carcinoma	7 (4)
*Clinical Stage* (*n*)	
Tumor classification (TNM 7th ed.)	
T2	3 (18)
T3	1 (6)
T4	13 (76)
Lymph node status (*n*)	
N2 (multistation)	11 (65)
N3	6 (35)
IIIA	2 (12)
IIIB	15 (88)
*Chemotherapy regimen* (*n*)	
Cisplatin/Docetaxel	17 (100)
*Clinical re-staging* (*n*)	
Tumor classification (*n*)	
T0	4 (23)
T1	1 (6)
T2	3 (18)
T4	9 (53)
Lymph node re-staging (*n*)	
N0	12 (70)
N2	3 (18)
N3	2 (12)

TNM Staging system 7th Edition used to classify tumors

All patients were initially staged with a total body CT scan, integrated ^18^F-FDG PET-CT scan, fibroscopy (FBS) and trans-bronchial nodal aspiration (TBNA), endoscopic bronchial/esophageal ultrasound (EBUS and EUS) and guided fine needle aspiration (LN FNA) for mediastinal lymph nodes staging. Brain MRI was performed only in case of suspicious brain metastases from the head CT. Other inclusion criteria were: performance score (PS) 0–1, < 70 years of age, > 3 months of life expectancy, presence of one measurable lesion according to RECIST criteria 1.1, normal organ and bone marrow function, FEV 1 (forced expiratory volume in the first second) > 50% of its predicted normal value. Exclusion criteria were: small-cell lung cancer (SCLC) diagnosis, prior neuropathy with neurotoxicity ≥2 (NCI/CTC), obstructive pneumonia, bronchial fistula, prior chemotherapy, prior radiotherapy to the thorax, > 10% weight loss in the last 6 months, stage-IIIA (T3 N1 or N2 < 3 lymph nodes without extracapsular involvement) or stage-IIIB NSCLC with supraclavicular and scalenic lymph nodes involvement, pleural and pericardial effusion, documented esophageal involvement in the entire thickness, involvement of cardiac ventricles, vertebral foramen and/or spinal marrow according to TNM Cancer Staging 7th edition. Patients with other malignancies in the 5 years before entering the study, except cured basal cell carcinoma of the skin and cured in situ carcinoma of the uterine cervix, or any other morbidity contraindicated for chemotherapy (e.g. heart diseases, active infections) were ineligible. We discussed all these cases in our multidisciplinary meeting called: Gruppo interdisciplinare patologia oncologica (GIPO).

**Fig. 1:**
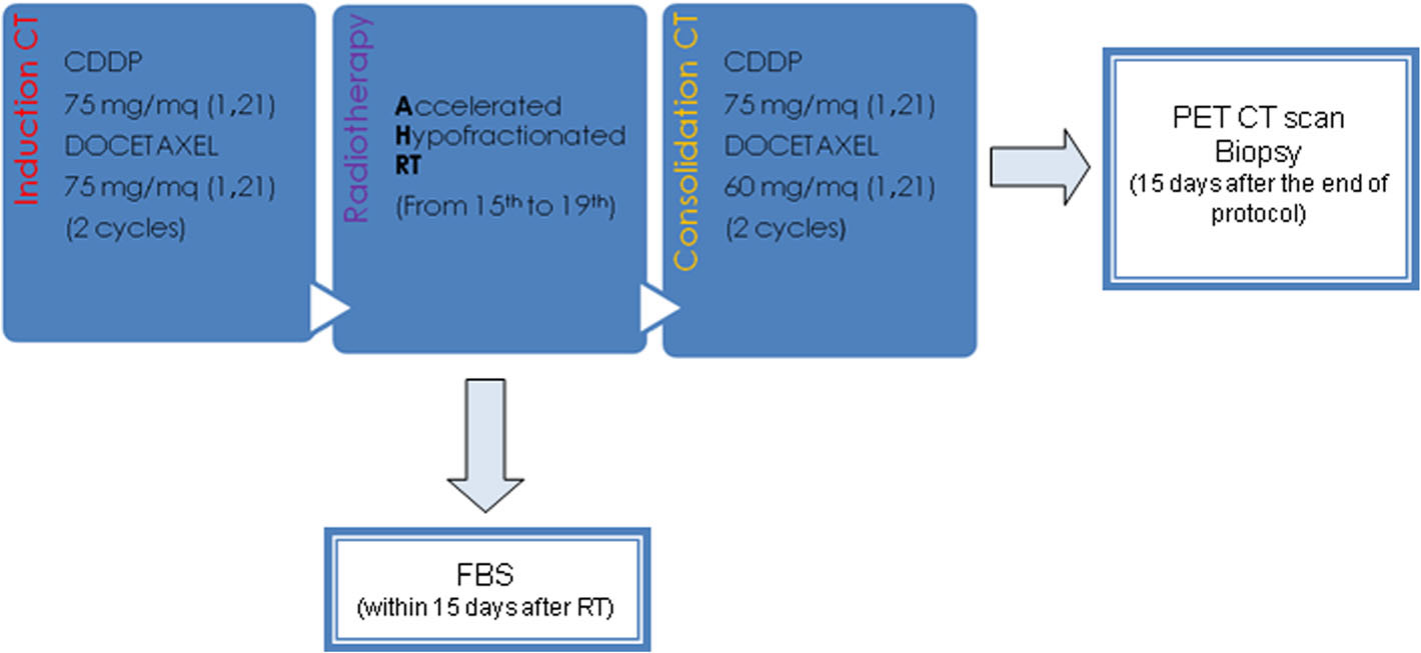
Trial design. This flow chart describes the scheduled chemotherapy and radiotherapy (RT) treatment. All patients underwent Fibroscopy (FBS) with nodal biopsy 15 days after the end of RT. The radiological evaluation was performed 15 days after the end of the protocol and after the biopsy with Positron Emission Tomography/Computed Tomography (PET/CT)

Patients received two courses of induction chemotherapy with cisplatin 75 mg/m^2^ and docetaxel 75 mg/m^2^ on day 1 every 21 days. After the second chemotherapy cycle, between days 15 and 19, patients underwent radiotherapy using helical tomotherapy (TomoTherapy Inc., Madison, WI, USA). After at least 15 days of radiotherapy, patients were administered two consolidation courses of chemotherapy with cisplatin 75 mg/m^2^ and docetaxel 60 mg/m^2^ on day 1 every 21 days (Fig. 1). The use of granulocyte-colony stimulating factor (*G*-*CSF) was allowed for secondary neutropenic* prophylaxis.

To perform radiotherapy, patients were immobilized in the supine position with their arms overhead using a Posirest-2 (CIVCO) prior to CT simulation scan. All patients underwent a planned CT scan with contrast agent injection (Brilliance Big Bore CT Philips, Crowley, UK). The chest CT simulation scan was acquired from the middle neck to the upper abdomen at time points 0, 30, 60 and 180 s during patients’ free breathing in order to simulate the respiratory movement. All non-fused phases of the CT simulation scan were used to contour the gross tumor volume (GTV), the clinical target volume (CTV) and the organs at risk (OaR) for radiotherapy planning. GTV encompassed all known primary tumors and involved lymph node locations. Additional manual margins were applied for microscopic tumor (CTV) and lymph node extension following the respiratory movements observed in the multiphase CT simulation scan images to create a non-isotropic margin. Daily set-up errors were corrected using image guided radiotherapy (IGRT) with ConeBeamCT. Finally, the OaR were also delineated, i.e. the esophagus, heart, spinal cord (with a 0.3-mm safety margin extension), lungs, total lung, liver, stomach and body*.* The Pinnacle treatment planning system (version 9.3) was used for contouring. Contours were transferred onto a Hi-Art tomotherapy treatment planning station for inverse planning calculation.

**Fig. 2:**
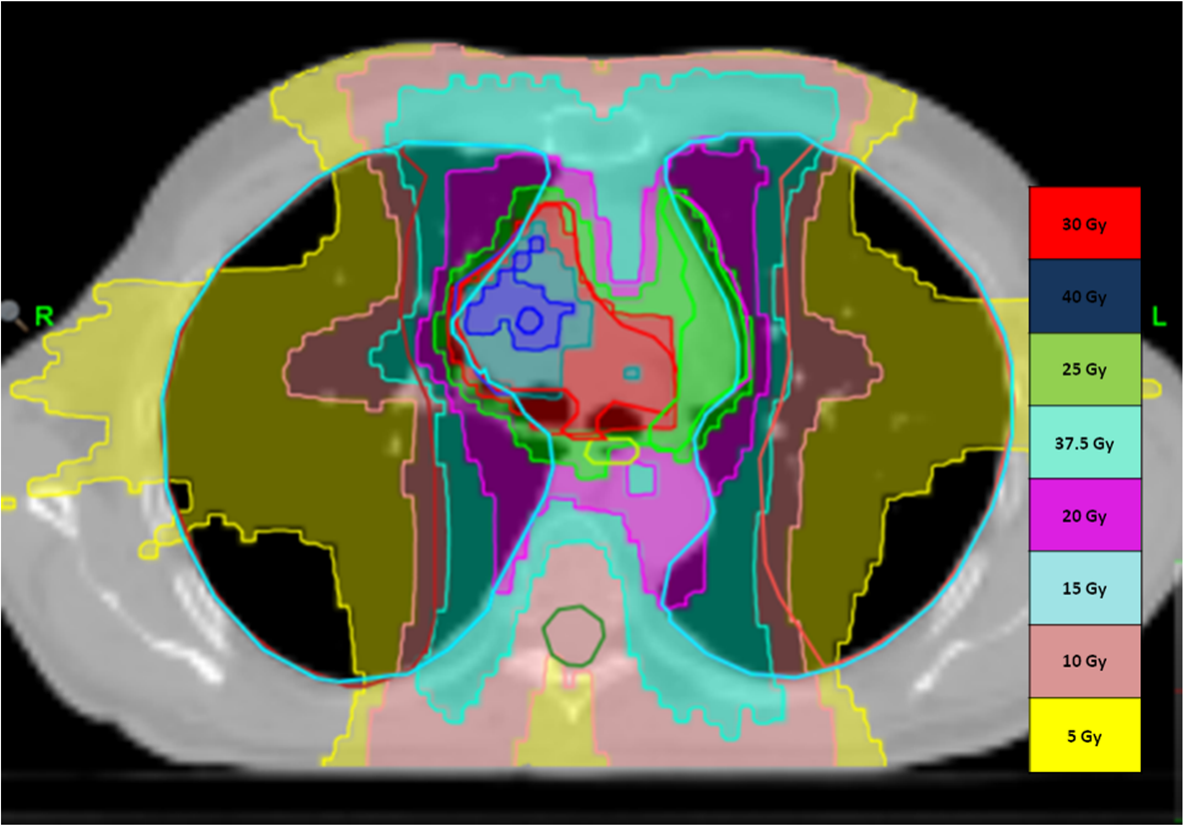
Example of dose distribution in a patient with stage-IIIB pulmonary adenocarcinoma after 2 induction chemotherapy cycles. The color legend DVH in the column on the right illustrates the dose distribution. Prescription dose was 30 Gy/5 daily fractions with a heterogeneous dose escalation of up to 40 Gy inside the primary tumor to simulate brachytherapy dose distribution. Prescription dose was 25 Gy/5 daily fractions with a heterogeneous dose escalation of up to 37.5 Gy inside the nodal tumor. The different colors show the following isodoses: red, 30 Gy; deep blue, 40 Gy; aqua, 37.5 Gy; green, 25 Gy; purple, 20 Gy; light blue, 15 Gy; pink, 10 Gy; yellow, 5 Gy

A dose of 30 Gy/5 daily fractions at the reference isodose (60–70%) was prescribed to the CTV, with an increasing inhomogeneous dose within the tumor of up to 40 Gy to simulate brachytherapy dose distribution [14, 15]. A dose of 25 Gy/5 daily fractions at the reference isodose (60–70%) was prescribed to the lymph nodes CTV, with an increasing inhomogeneous dose of up to 37.5 Gy. We used a SBRT-like dose prescription to increase dose heterogeneity within the target (Fig. 2). The prescribed dose at 70% isodose allowed for the delivery of a high dose to the GTV, sparing as much normal lung tissue as possible. The OaR dose volume histograms (DVHs) were converted into a 2-Gy equivalent dose; we applied the dose constraints recommended by the literature data [16], while maintaining the OaR doses below the conventional fractionation values (Table 2a-b).

**Table 2:** a, b Relevant normal tissue dosimetric parameters for organs at risk (OaR)

a: dosimetric values for total lung						
OaR	Total lung (α/β 3.1)					
	V5	V10	V20			
Mean	1337	873	514 (13%)			
Max	2502	1551	1241 (27%)			
Min	832	422	191 (5%)			
b: dosimetric values for esophagus, cord and heart						
OaR	Esophagus		Cord		Heart	
	α/β 4		α/β 3		α/β 3	
	NTD2 mean	NTD2 max Gy	NTD2 mean Gy	NTD2 max Gy	NTD2 mean Gy	NTD2 max Gy
Mean (Gy)	10	22	3	7	4	23
Max (Gy)	43	72	12	18	41	3
Min (Gy)	1	4	0	0	0	80
V40(cc)	0	4	0	0	7	102
V55(cc)	0	0	0	0	0	6

V5, V10, V20 = total lung volume (cc) receiving 5, 10, 20 Gy, respectively

In the V20 column, below cc value is reported also percent value

NTD2 = normal tissue dose 2 Gy equivalent reported with maximum and mean dose for all OaR; V40 and V55 = organs at risk (cc) receiving 40 and 55 Gy, respectively

All treatments were performed using helical tomotherapy. Restaging procedures included integrated ^18^F-FDG PET-CT scan and EBUS/EUS FNA performed after at least 2 weeks of end of the protocol program. The mediastinal lymph node stations positive at staging were approached again with TBNA or FNA to confirm disease persistence or down-staging. After the end of the protocol, patients underwent a follow-up evaluation with a total body CT scan and laboratory examinations every 12 weeks. PET scan was performed in case of suspicion of disease progression.

The primary endpoint of the study was to determine the objective tumor response rate (RR) according to RECIST criteria 1.1. Secondary endpoints were acute and late side-effects, local progression-free survival (PFS), metastasis-free survival (MFS) and overall survival (OS).

This study was approved by the Ethics Committee IRST IRCCS AVR with approval number 331 05/11/2008, was also conducted in accordance with the Declaration of Helsinki and all patients signed the informed consent.

### Statistical analysis

Objective RR [complete (CR) and partial response (PR)] was the primary endpoint of our study. The sample size was calculated using a minimax 2-stage Simon design. A 70% objective RR was considered acceptable for further testing of the experimental treatment, whereas a 50% objective RR was ruled out as futile. Using alpha and beta values of 0.05 and 0.20, respectively, at least 13 of the 29 patients were required to have obtained CR or PR in the first stage before moving on to the second stage. As an additional 14 patients were enrolled in the second stage, at least 24 of the total 43 patients were required to have reached CR or PR in the second stage for the combination to be considered sufficiently active to undergo further testing.

Unfortunately, after 36 months’ recruitment, the protocol committee decided to close the study due to a poor accrual rate, as only 17 evaluable patients had been enrolled. Despite the low statistical power of the study, the authors decided to present their results.

Acute and late toxicities were recorded according to NCI common terminology criteria for adverse events (CTCAE) version 3.0 [17] after patients had completed the protocol. In particular, dyspnea was measured according to patient-reported shortness of breath, grade of exertion, limitation of daily activities, as reported in CTCAE 3.0.

RR was evaluated as the proportion of patients experiencing reduction in tumor burden according to RECIST criteria 1.1. Progression-free survival (PFS) was calculated as the time from enrollment until disease progression or death, whichever came first. Local PFS (LPFS) was defined as the time during and after treatment with stable disease. Metastasis-free survival (MFS) was defined as the time from accrual to disease progression. OS was calculated according to the Kaplan-Meier method as the time from diagnosis until death or last recorded follow-up visit. All statistical analyses were performed using SAS Statistical software (version 9.3, SAS Institute Inc., Cary, NC, USA).

## Results

From January 2009 to January 2012, 23 consecutive eligible patients were enrolled in the trial, of whom only 17 (74%) completed the protocol. In particular, of the 6 patients unable to complete the study, 2 had an allergic reaction to docetaxel and received a modified chemotherapy schedule, 1 developed tumor abscess complicated in lung infection after chemotherapy and received radiotherapy about 8 months later for local progression of disease, 1 underwent paroxysmal atrial fibrillation after the first course of chemotherapy, 1 died after induction chemotherapy for unspecified causes, and 1 developed G2 pulmonary hemorragie after induction chemotherapy.

Twenty-eight days (range 18–45) was the average time from the end of radiotherapy to the start of the third course of chemotherapy. Twenty-nine percent of patients received 75% of the planned dose of chemotherapy, whereas 47% of chemotherapy courses were administered using granulocyte-colony stimulating factor (*G*-*CSF)* as secondary neutropenic prophylaxis.

All patients completed the radiotherapy schedule. Table 2a-b collects the information about the OaR.

Acute grade (G) side-effects due to chemotherapy were G4 neutropenia (70%) and G4 leucopenia (24%), G3 paresthesie (24%), G3 lung infection (4%), whereas no severe acute side-effect was recorded during radiotherapy.

All side-effects are listed in Table 3. We analyzed the results of pulmonary function test and noticed that in the acute period there was a reduction in FEV1 and DLCO to a value of max 20 points related to a moderate ventilatory deficit and a value of max 28 points reduction related to a moderate capillary socket diffusion, respectively. As for late toxicity, we noticed that 13 patients had recovered from acute damage with normalization of FEV1 and DLCO values, whereas 4 patients who had experienced G3 pneumonitis had maintained their functional damage. Protocol-associated toxicity was particularly critical for chemotherapy. We reported 1 case of death after 1 cycle of chemotherapy induction for unspecified cause, and 5 cases of study drop-out due to induction chemotherapy toxicity.

**Table 3:** Acute and late toxicity

Toxicity (NCI CTCAE v 3.0)	Grade 1		Grade 2		Grade 3		Grade 4		Grade 5	
	*n*	%	*n*	%	*n*	%	*n*	%	*n*	%
Acute (*n* = 23)										
Neutropenia	–	–	2	12	–	–	12	70	–	–
Leucopenya	–	–	–	–	5	29	4	24	–	–
Anemy	–	–	2	12	–	–	–	–	–	–
Mucosyte	–	–	2	12	–	–	–	–	–	–
Creatinine	–	–	3	18	–	–	–	–	–	–
Paresthesie	–	–	10	59	4	24			–	–
Dyspnea	13	76	2	12	–	–	–	–	–	–
Cough	10	59	3	18	–	–	–	–	–	–
Fever	–	–	–	–	–	–	–	–	–	–
Esophagitis	5	29	3	18	–	–	–	–	–	–
Fatigue	–	–	3	18	2	12	–	–	–	–
Pneumonitis	11	65	3	18	–	–	–	–	–	–
Cardiac arrhythmia					1	4				
Unspecified causes									1	4
Pulmonary Hemorragie			1	4						
Allergic reaction					2	12				
Pulmonary abscess										
Late (*n* = 17)										
Dyspnea	3	18	1	6	4	24	–			
Cough	–	–	1	6	–	–	–	–		
Pneumonitis	7	41	–	–	–	–	–	–		

Data presented as number (*n*) and percentage of patients who developed toxicity. Acute toxicity was calculated for all the 23 patients enrolled

The treatment yielded radiologically and hystologically proven disease down-staging in 7 (41%) patients; the remaining 10 (59%) patients had stable disease. The 41% of patients experienced down-staging showed a longer OS of 86.7 months (95% CI 7.8-not reached) vs. 12.5 months (95% CI 6.2–27.1), a lower PFS of 6.2 months (95% CI 5.6–50.8) vs. 8.7 months (95% CI 5.3–10.1), and a lower MFS 6.2 of months (95% CI 5.6–50.8) vs. 9.7 months (95% CI 5.3-not reached) in patients who experienced stable disease.

Among the RR with down-staged patients, 18% reached CR, 65% had mediastinal lymph node down-staging with 70% N0, 18% N2 and 12% N3 persistent, and 41% obtained tumor shrinkage, as shown in Table 1. A 76% tumor RR was achieved according to RECIST criteria 1.1.

Forty-one percent of patients had tumor recurrence, of whom 23% in the same radiotherapy field and 18% outside the field, recorded during follow up time.

**Fig. 3:**
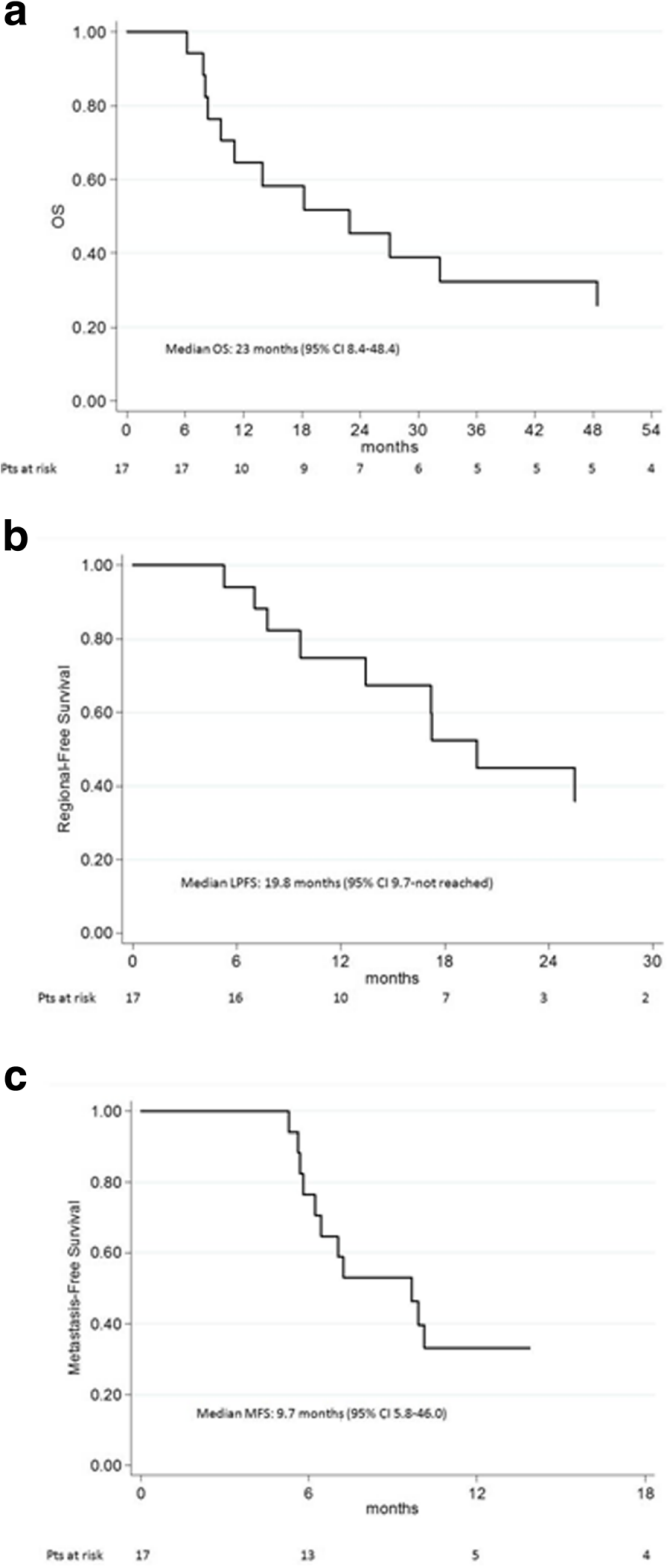
**a**, **b**, **c** Overall survival (OS) (**a**), local progression-free survival (LPFS) (**b**), metastasis-free survival (MFS) (**c**). OS was calculated according to the Kaplan-Meier method as the time from diagnosis until death or last recorded follow-up. *Pts* = patients

After a median follow-up of 87 months (range 6–87), median LPFS was 19.8 months (95% CI 9.7-not reached) and median MFS was 9.7 months (95% CI 5.8–46.0) with a median OS of 23 months (95% CI 8.4–48.4), as shown in Fig. 3a, b, c.

## Discussion

The novel approach of this protocol was the delivery of AHR to the tumor site and involved mediastinal lymph nodes. To our knowledge, this is the first protocol employing AHR with chemotherapy for the treatment of locally advanced NSCLC. The choice to combine accelerated hypofractionation radiotherapy and sequential chemotherapy instead of concomitant chemotherapy, was driven by the opportunity to avoid relevant acute toxicity. We used IMRT in tomotherapy and daily IGRT to deliver the treatment, in order to obtain disease down-staging and assess the efficacy of the proposed RT dosage schedule of 30 Gy/5 daily fractions to the primary tumor and the mediastinal nodes.

In Johnson et al.’s [18] phase-II trial, patients with locally advanced NSCLC were treated with induction chemotherapy with cisplatin and vinblastine, then evaluated for surgery or healing radiotherapy for a total dose of 54 Gy in 27 fractions: median OS was 12 months, and survival rates at 12, 24 and 36 months were 54, 39 and 11%, respectively. Furthermore, Perez et al. [19] published the final results of their study comparing different treatment schedules and the best results were obtained with daily bi-division, for a total dose of 69.6 Gy, with survival rates at 12 and 36 months of 58 and 20%, respectively. The combination of weekly paclitaxel, carboplatin and TRT (63 Gy) consolidation was also associated with optimal outcome but with important toxicity [20]. The RTOG 0617 study reported a median OS of 28.7 months for patients who received standard chemo-radiotherapy, which is higher than 23.0 months reported by our study. The reason for this could be traced to patient selection: in our study 88% of patients were staged IIIB, whereas only 34% of patients were staged IIIB in the RTOG 0617 study. The fact that IIIB-stage patients reported a worse prognosis than IIIA-stage patients may explain the difference in OS. Past studies obtained a similar OS rate to DART-bi, by exploiting the concept of dose differentiation (DART-bi) according to tumor size [21]. Moreover there are some retrospective studies about hypofractionation intensity modulated radiotherapy with similar patient selection, fractionation, range of prescribed dose, and technique, that report different OS data [22, 23]. Swanick et al. reported a retrospective analysis of patients with non-operable NSCLC (all stages) treated with IMRT-SIB in 15 fractions recording a median OS of 9 months. Westover et al. and Pollom applied the same schedule of treatment to all stages of disease, reporting a median OS of 12.5 months and 15.1 months, respectively [23, 24]. The differences reported in these studies may be related to patient selection and prescribed dose range. Better OS results were obtained in the SOCCAR study with an OS of 24.3 months for inoperable patients treated with radical intent and a schedule treatment of 55 Gy/20 fractions [25]. Another relevant radiotherapy scheme, 2.75 Gy/24 fractions associated to concurrent chemotherapy yielded an impressive median OS rate of 31.5 months [26].

Notwithstanding that stereotactic treatment on lung cancer has demonstrated the feasibility of AHR [12], one of the major concerns about its use is the potential toxicities [27]. A systematic review of the literature by Kaster et al. [28] on hypofractionation in locally advanced NSCLC reported improving outcomes in stage-III NSCLC with some schedules of AHR in systematic concurrent chemotherapy. The authors analyzed AHR with a dose ranging 45–85.5 Gy in 15–35 fractions, with a dose per fraction ranging 2.3–3.5 Gy, and concluded that AHR may improve local control, increasing the biological effective dose.

In our study, however, the dose delivered to the primary tumor ranged 6–8 Gy/fraction inhomogeneous dose (increasing within the tumor), and the dose delivered to the involved mediastinal nodes ranged 5–7.5 Gy/fraction in 5 daily fractions (increasing the inhomogeneous dose within the involved mediastinal nodes). The acceptable toxicity profile of the patients determined the feasibility of the proposed treatment schedule in combination with sequential chemotherapy. As reported in other studies [28], especially Cannon et al. [29], patient toxicity was correlated with the dose delivered to the OaR in the majority of cases. In their phase-I study using AHR for locally advanced NSCLC, Cannon et al. treated patients without concurrent chemotherapy, showing that hypofractionation of up to 63.25 Gy in 25 fractions (2.5 Gy/fraction) is well tolerated when strict normal tissue constraints are maintained. The most severe reported toxicity correlated to the total prescribed dose was G4–5 in the proximal bronchial tree and surrounding vasculature. Timmerman et al. [30] described a high potential damage of the central structures, such as complications due to the use of stereotactic body radiotherapy (SBRT) for the treatment of early stage NSCLC.

Hence, we also prescribed 30 Gy close to the central region positioning the inhomogeneous dose point of 40 Gy away from the bronchial structure so as to spare the bronchial tree (see Fig. 2) and avoid any G4–5 bronchial and vascular toxicity. We recorded no G4–5 toxicity for any of the OaR, probably as a result of the dose constraints because IMRT can considerably reduce the irradiated volume of the OaR while maintaining adequate dose coverage to the target, as also reported by Liu et al. [31] and also thanks to IGRT. Our data revealed G4 neutropenia and leucopenia related to chemotherapy, as 29% of chemotherapy was administered at a reduced dose, in particular for docetaxel. At the end of the protocol, 41% of patients had radiologically and histologically proven disease down-staging, whereas 59% had stable disease with 76% RR according to RECIST criteria 1.1. In particular, we recorded 18% of CR, 65% of involved lymph nodes down-staging, and 41% of primary tumor down-staging. The mediastinal downstaging is an important objective to reach for radiation oncologist and probably this fractionation could be considered as the first step to increase further the dose prescription to involved mediastinal nodes.

Indeed, one of the major issues in the treatment of locally advanced stage-IIIB NSCLC is locoregional control improvement. A relatively recent meta-analysis [32] of six randomized trials comparing concomitant vs. sequential chemoradiotherapy has shown that the former has a benefit in terms of locoregional progression (*p* = 0.01) and a lower rate of locoregional progression of disease at 5 years (28.9% vs. 35%), whereas no difference was found in terms of distant metastasis (*p* = 0.69). The Auperin study concluded that the concomitant approach yields better OS than sequential chemoradiotherapy due to better LC, which is achieved by radio-sensitizing the tumor with simultaneous application of chemotherapy. In our cohort, stage-IIIB NSCLC patients (88%) were able to undergo AHR with this treatment schedule, as adequate locoregional control sequential to chemotherapy had improved.

The literature reports that a high dosage per fraction, such as 3 Gy concurrent to chemotherapy with two agents, could lead to severe toxicity, in particular with the use of 3-dimensional conformal techniques [33]. We administered 6 Gy/fraction without reporting any G4–5 toxicity in normal tissue using intensity modulated arch therapy in tomotherapy in order to spare the OaR. Avoiding the contour of isotropic margin to the GTV, we irradiated a limited volume of normal pulmonary tissue. An important issue in our study was dosage appropriateness. We used BED_10_ at the isocenter as a function of tumor size (mean tumor volume was 150 cc) [34]. The calculated mean dose to the tumor was 74 Gy. With regard to the nodal volume, we used BED_10_ at the isocenter with a calculated mean dose of 54 Gy. Similarly to the literature data, our results confirmed the efficacy of the delivered doses.

The CHART study introduced continuous AHR in a randomized trial that showed better survival than conventional fractionation [35]. The final results of the CHARTWEL trial have laid down the basis for dose intensification trials for locally advanced NSCLC [36]. More recently, van Baardwijk published the results of a median OS of 25 months in a phase-II trial based on individualized accelerated radiotherapy concurrent with chemotherapy [37].

In the RTOG 0617 protocol a high number of deaths were reported in the arm receiving 74 Gy/fraction, probably due to radiotherapy-related normal tissue toxicity, even if the toxicity profile of these patients was the same as of those in the 60 Gy/fraction arm [11]. To avoid severe G4–5 toxicity, we used IMAT with tomotherapy, administering radiotherapy between chemotherapy cycles (between days 15 and 19 after the second chemotherapy cycle).

Recently, the PACIFIC study [38] reported the best PFS with radiochemotherapy treatment, concomitant or sequential followed by an anti PD-L-1 antibody, Durvalumab, in patients with inoperable locally advanced NSCLC. The median PFS was 16.8 months (95% confidence interval [CI], 13.0 to 18.1) with Durvalumab vs. 5.6 months (95% CI, 4.6 to 7.8) with placebo (stratified hazard ratio for disease progression or death, 0.52; 95% CI, 0.42 to 0.65). The OS rate at 24 months was 66.3% (95% CI, 61.7 to 70.4) in the durvalumab group, as compared with 55.6% (95% CI, 48.9 to 61.8) in the placebo group (two-sided *p* = 0.005). This is the future of the treatment of locally advanced NSCLC, considering also that many data suggest that the best association with immunotherapy could be hypofractionation [39].

Among the limitations of our study are the fact that it was designed before the use of immunotherapy in this pateint setting, the long period of accrual, restricted cohort of patients, i.e. Twenty-three enrolled patients, of whom only 17 were eligible, which was probably the greatest limitation in consideration of the results obtained for toxicity, PFS and OS. Furthermore, the accrual process was complicated as stage-IIIB lung cancer often hides stage-IV metastases and the trial was closed early, prior to first planned interim analysis, due to poor accrual. Another limitation of the study was the lengthy time between the last RT fractionation and the start of the last two chemotherapy cycles. The calculated period was an average of 28 days, even though a period of up to 15 days had been established, according to the protocol. One reason for this delay was that FBS was performed later than the established period due to organizational issues. Another drawback of the study was that brain CT was routinely performed in place of brain MRI.

## Conclusions

In conclusion, chemo-radiotherapy is the gold standard in the treatment of unresectable stage-III locally advanced NSCLC. Since this kind of treatment is characterized by high toxicity, it is pivotal to counterbalance good results with acceptable toxicity. With similar rates of efficacy, a shorter treatment time would reduce overall treatment costs and improve patient compliance. As no high-grade radiotherapy-related toxicity was observed, the dose delivered to the tumor and lymph nodes might be increased. Finally, it is important to underline that the new technologies now allow for high radiation dose delivery, which can be associated with new targeted therapies.

## Data Availability

All data generated or analyzed during the current study are available from the corresponding author on reasonable request.

## Abbreviations

3D-CRT: 3-dimensional conformal radiotherapy
AHR: Accelerated hypofractionated radiotherapy
CTV: Clinical target volume
DVHs: Dose volume histograms
EBUS: Endoscopic bronchial esophageal ultrasound
FEV: Forced expiratory volume
*G*-*CSF*: *G*ranulocyte-colony stimulating factor
GTV: Gross tumor volume
IGRT: Image guided radiotherapy
IMAT: Intensity modulated arc therapy
LN FNA: Guided fine needle aspiration
MFS: Metastasis-free survival
NSCLC: Non-small-cell lung cancer
OaR: Organs at risk
OS: Overall survival
PFS: Progression-free survival
RR: Response rate
SCLC: Small-cell lung cancer
